# Performance Study of Distance-Weighting Approach with Loopy Sum-Product Algorithm for Multi-Object Tracking in Clutter

**DOI:** 10.3390/s21072544

**Published:** 2021-04-05

**Authors:** Pranav U. Damale, Edwin K. P. Chong, Tian J. Ma

**Affiliations:** 1 Department of Electrical and Computer Engineering, Colorado State University, Fort Collins, CO 80523-1373, USA; edwin.chong@colostate.edu; 2 Sandia National Laboratories, Albuquerque, NM 87123, USA; tma@sandia.gov

**Keywords:** multiobject tracking, high-clutter tolerant multiobject tracking, probabilistic data association (PDA), joint probabilistic data association (JPDA), loopy sum-product algorithm (LSPA)

## Abstract

In this paper, we explore the performance of the distance-weighting probabilistic data association (DWPDA) approach in conjunction with the loopy sum-product algorithm (LSPA) for tracking multiple objects in clutter. First, we discuss the problem of data association (DA), which is to infer the correspondence between targets and measurements. DA plays an important role when tracking multiple targets using measurements of uncertain origin. Second, we describe three methods of data association: probabilistic data association (PDA), joint probabilistic data association (JPDA), and LSPA. We then apply these three DA methods for tracking multiple crossing targets in cluttered environments, e.g., radar detection with false alarms and missed detections. We are interested in two performance metrics: tracking accuracy and computation time. LSPA is known to be superior to PDA in terms of the former and to dominate JPDA in terms of the latter. Last, we consider an additional DA method that is a modification of PDA by incorporating a weighting scheme based on distances between position estimates and measurements. This distance-weighting approach, when combined with PDA, has been shown to enhance the tracking accuracy of PDA without significant change in the computation burden. Since PDA constitutes a crucial building block of LSPA, we hypothesize that DWPDA, when integrated with LSPA, would perform better under the two performance metrics above. Contrary to expectations, the distance-weighting approach does not enhance the performance of LSPA, whether in terms of tracking accuracy or computation time.

## 1. Introduction

Since probabilistic data association (PDA) was introduced for tracking in a cluttered environment [1], engineers have been trying to optimize the data association (DA) technique for implementing in a Kalman filter (KF) [2] tracker. With the inception of the sum-product algorithm (SPA) [3], a new graphical approach for KF tracking was developed that is more efficient in dealing with the complex problem of multiple target tracking (MTT). To develop a robust algorithm that is also scalable for tracking multiple objects in clutter, in this paper we examine the performance of the distance-weighting probabilistic data association (DWPDA) [4] in conjunction with the loopy sum-product algorithm (LSPA) [5].

The problem of DA is finding the correspondence between the targets and the measurements of uncertain origins. There are several approaches to tackle this problem, such as the nearest neighbor (NN), PDA, and joint probabilistic data association (JPDA) [1,6,7]. The NN approach is one of the easiest and uses at any time only the nearest measurement to the predicted measurement as if it were the one originated from the target of interest [6]. The NN approach is suboptimal and can only work well in case of widely spaced targets, accurate measurements, and few false alarms. Finding some association between all the targets and measurements is computationally expensive. Therefore a gate is formed around the predicted measurement based on some predefined threshold called the validation region. The PDA algorithm obtains the probability of each measurement lying inside the validation region as being the correct measurement and updates the state estimates according to an appropriately modified tracking filter, called PDAF [1]. One major drawback of the PDA algorithm is that it treats every measurement inside the validation region of a target of interest as if it were originated from the said target or as a Poisson-distributed false alarm. PDA does not take into account the possibility that a validated measurement for one target might be a measurement originated from another nearby target. The JPDA algorithm improves this deficiency of the PDA algorithm by computing the association probabilities for the validated measurements from the joint likelihood functions corresponding to all feasible joint events such that no more than one measurement originates from each target [7]. However, with an increasing number of targets and/or clutter, the JPDA algorithm becomes impractical for real-time applications due to its combinatorial complexity because it considers all feasible joint events of measurements to targets to obtain the joint association probabilities [8].

PDA and JPDA are zero-scan algorithms, meaning that all hypotheses are combined after computation of the probabilities, for each target at each time step [1,7]. Alternatively, multiple hypothesis tracking (MHT) is a deferred decision logic algorithm. In the case of a conflicting target-measurement association, the MHT algorithm formulates alternative data association hypotheses instead of choosing the best-combined hypothesis. The ambiguities between the alternative hypotheses are resolved using the measurements that arrive later into the future [9]. JPDA is shown to produce reasonable results compared to the computationally expensive multi-scan MHT algorithm [7,8,9,10]. Both the PDA and the JPDA algorithms utilize known targets for forming validation gates in the measurement space to compute the posterior probabilities and are therefore categorized as target-oriented approaches. There exist measurement-oriented algorithms, such as the one described in [11] and others, where hypotheses are formed for each measurement to have originated from a known target, a new target, or clutter. Both sets of algorithms have shown to yield an equivalent expression for posterior probabilities with appropriate assumptions [7,11,12].

As we realize that calculating DA probabilities for MTT is a process that involves a complicated global function of many variables that can be broken down into a product of local functions each of which depends on a subset of the variables, we look for alternate methods that can exploit this trait. SPA is one such method that operates in a factor graph [13] and attempts to compute, either exactly or approximately, various marginal functions associated with the global function [3]. SPA operates by passing messages, called beliefs, between the nodes of a factor graph, i.e., variables and local functions, that involve summations and products of factors. Implementation of a factor graph for the MTT DA problem requires a loopy sum-product solution which is neither guaranteed to converge nor produce the correct marginal functions if convergence occurs. A simultaneous target-oriented and measurement-oriented factor graph formulation of the DA problem has been shown that is guaranteed to converge and results in accurate association probabilities [5,14]. A simplified implementation of LSPA results in a significant reduction in computational complexity without loss of accuracy [14,15], which makes LSPA more appealing than PDA or JPDA for DA.

In recent years, the use of LSPA for DA in MTT has been gaining traction. A belief-propagation approach to multi-target multi-sensor tracking is proposed in [16] by formulating a detailed factor graph where every single target and data-association variable is modeled as an individual node. To tackle the problem of tracking an unknown number of targets, where targets appear and disappear, an LSPA-based method is proposed in [17] that creates augmented target states for keeping track of existing and non-existing targets. A more comprehensive derivation of the message-passing algorithm for multi-sensor multi-target tracking is given in [18,19]. An extension of the LSPA-based MTT framework for target estimation is given in [20] that exploits additional target information provided by a classifier. All these efforts highlight the potential of SPA for solving the complicated problem of DA in MTT.

The data association probabilities obtained using PDA are a crucial part of the beliefs being passed between nodes during the convergence of LSPA. Modifying the DA probabilities according to a weighting scheme based on distances between the predicted and validated measurements has been shown to enhance the tracking accuracy of PDAF while tracking a single target in a densely cluttered environment [4]. In this paper, we want to explore whether this distance-weighting approach for PDA, when integrated with LSPA, would improve the performance of LSPA even further. To evaluate this possibility, we formulate a distance-weighting LSPA (DWLSPA) and compare its performance in terms of tracking accuracy and computation time against DWPDA, JPDA, and LSPA for tracking multiple targets crossing at a small angle in different density clutter. Our results turn out to be contrary to expectations.

The main contribution of this performance study is to explore the idea of modifying one of the building blocks for a state-of-the-art data-association algorithm [15] for multi-target tracking and to compare the tracking accuracy of the modified algorithm to that of the original algorithm. The distance-weighting modification analyzed in this paper is based on a recent successful implementation of a similar modification to one of the earliest data-association frameworks for tracking targets in cluttered environments [4]. In doing so, we develop the mathematical formulation for each data-association filter being considered and evaluate its performance in terms of tracking accuracy and computation time over a wide range of easy-to-replicate multi-object-tracking scenarios.

We introduce our notations and the target tracking system in Section 2. In Section 3, we describe the problem of PDA, DWPDA, JPDA, and LSPA. We present simulation results for these methods and our analysis in Section 4. In Section 5, we conclude the paper with our observations and final remarks regarding the performance of these methods.

## 2. Target Tracking Dynamic System Model and Assumptions

We describe the classic data association problem in which a single sensor surveils a large number of targets. The number of targets under surveillance is assumed to be known and is denoted by $N_{T}$. The measurements are comprised of possible target detections and false alarms. A target is detected with a known probability of detection $P_{D}$ and is independent of time. The false alarms, modeled according to the Poisson point process with a known spatial density λ, are uniformly distributed in the measurement space. A validation region, with the threshold γ corresponding to certain gate probability $P_{G}$, is set up at every sampling time around the predicted measurement and possibly several measurements fall in it. Each algorithm differs in how these measurements are used (or not) in the estimation of the state of the target. We assume that each target can generate at most one measurement and each measurement can have only one source.

We denote by $x_{i}\left(k\right)$, $i\in \{1,\ldots ,N_{T}\}$, the state of *i*-th target of dimension $n_{x}$ at time *k*. The complete set of target states at time *k* is denoted by $X\left(k\right)=(x_{1}\left(k\right),\ldots ,x_{N_{T}}\left(k\right))$. At time *k*, the total number of measurements is denoted by M(k). We denote by $z_{j}\left(k\right)$, j∈{1,…,M(k)}, the value of *j*-th measurement of dimension $n_{z}$. The complete set of measurements at time *k* is denoted by $Z\left(k\right)=(z_{1}\left(k\right),\ldots ,z_{M(k)}\left(k\right))$, and the complete set of measurements up to and including time *k* is denoted by $Z^{k}=\left(Z\left(1\right),\ldots ,Z\left(k\right)\right)$.

The state and measurement equations are assumed linear with additive zero-mean white noise with known covariances. The state of target *t* evolves in time according to the equation

(1) $$x_{t}\left(k+1\right)=F\left(k\right)x_{t}\left(k\right)+G\left(k\right)u\left(k\right),$$

and the true measurement for target *t* is given by

(2) $$z_{t}\left(k\right)=H\left(k\right)x_{t}\left(k\right)+w\left(k\right)$$

where u(k) and w(k) are zero-mean mutually independent white Gaussian noise sequences with known covariances Q(k) and R(k), respectively. Functions F(k),G(k), and H(k) are known matrices for state transition, noise gain, and sensor, respectively. The past information (through time k−1) about the target *t* is assumed to be known and summarized approximately by the Gaussian posterior

(3) $$p\left[x_{t}\left(k-1\right)|Z^{k-1}\right]=N\left[x_{t}\left(k-1\right);{\hat{x}}_{t}\left(k-1|k-1\right),P_{t}\left(k-1|k-1\right)\right]$$

where ${\hat{x}}_{t}\left(k-1|k-1\right)$ and $P_{t}\left(k-1|k-1\right)$ are the state estimate and covariance for target *t*.

## 3. Algorithm Description

### 3.1. Probabilistic Data Association Filter

The PDA algorithm calculates the association probabilities for each validated measurement at the current time for the target of interest. PDA assumes that all the validated measurements are generated by either the target of interest or clutter. The association probabilities are used for calculating the mean squared error (MSE) estimate and covariance of the target’s state. An appropriately modified KF, called PDAF, is used to account for the uncertainty of origins for the validated measurements while estimating the state of the target. The algorithm can be given as follows.

#### 3.1.1. Prediction

The prediction of the state and measurement of target *t* at time *k* is done as in the KF, i.e.,

(4) $$\begin{array}{cc} {\hat{x}}_{t}\left(k|k-1\right) & =F\left(k-1\right){\hat{x}}_{t}\left(k-1|k-1\right) \end{array}$$

(5) $$\begin{array}{cc} {\hat{z}}_{t}\left(k|k-1\right) & =H\left(k\right){\hat{x}}_{t}\left(k|k-1\right). \end{array}$$

The covariance of the predicted state for target *t* is

(6) $$P_{t}\left(k|k-1\right)=F\left(k-1\right)P_{t}\left(k-1|k-1\right)F{\left(k-1\right)}^{\prime }+G\left(k-1\right)Q\left(k-1\right)G{\left(k-1\right)}^{\prime }.$$

Here, ${\hat{x}}_{t}\left(k-1|k-1\right)$ and $P_{t}\left(k-1|k-1\right)$ are available from Equation (3). The innovation covariance of the target *t* (for the correct measurement) is

(7) $$S_{t}\left(k\right)=H\left(k\right)P_{t}\left(k|k-1\right)H{\left(k\right)}^{\prime }+R\left(k\right).$$

#### 3.1.2. Measurement Validation

The validation region for target *t* at time *k* is the elliptical region given by

(8) $$V_{t}\left(k,\gamma \right)=\left\{z\in Z\left(k\right):{\left[z-{\hat{z}}_{t}\left(k|k-1\right)\right]}^{\prime }S_{t}{\left(k\right)}^{-1}\left[z-{\hat{z}}_{t}\left(k|k-1\right)\right]\leq \gamma \right\}.$$

Here, γ can be obtained according to

(9) $$V\left(k\right)=c_{n_{z}}{\left|\gamma S\left(k\right)\right|}^{1/2}$$

where V(k) is the volume of the validation region given by Equation (8) and $c_{n_{z}}$ is the volume of the unit hypersphere of dimension $n_{z}$ [12]. The validated measurements for target *t* according to Equation (8) are

(10) $$Z_{t}\left(k\right)\overset{\Delta }{=}{\left\{z_{i}\left(k\right)\right\}}_{i=1}^{m_{t}\left(k\right)}$$

where $m_{t}\left(k\right)$ is the number of validated measurements for target *t* at time *k*.

#### 3.1.3. Data Association Probabilities

The association probability $\beta_{i}^{t}$ for each validated measurement of target *t* is obtained as

(11) $$\beta_{i}^{t}\left(k\right)=\left\{\begin{array}{c} \frac{L_{i}\left(k\right)}{1-P_{D}P_{G}+\sum_{j=1}^{m_{t}\left(k\right)}L_{j}\left(k\right)}\;i=1,\ldots ,m_{t}\left(k\right)\\ \frac{1-P_{D}P_{G}}{1-P_{D}P_{G}+\sum_{j=1}^{m_{t}\left(k\right)}L_{j}\left(k\right)}\;i=0 \end{array}\right.$$

where i=0 indicates probability of associating none of the validated measurements to the target. In Equation (11), $L_{i}\left(k\right)$ the likelihood ratio (LR) of measurement $z_{i}\left(k\right)$ originating from the target *t* vs. from clutter and is obtained as

(12) $$L_{i}\left(k\right)\overset{\Delta }{=}\frac{N\left[z_{i}\left(k\right);{\hat{z}}_{t}\left(k|k-1\right),S_{t}\left(k\right)\right]P_{D}}{\lambda },\;z_{i}\left(k\right)\in Z_{t}\left(k\right).$$

#### 3.1.4. State Estimation

The state estimation for target *t* is done according to PDAF by

(13) $${\hat{x}}_{t}\left(k|k\right)={\hat{x}}_{t}\left(k|k-1\right)+W_{t}\left(k\right)v_{t}\left(k\right)$$

where the combined innovation for target *t* is

(14) $$v_{t}\left(k\right)=\sum_{i=0}^{m_{t}\left(k\right)}\beta_{i}^{t}\left(k\right)v_{i}^{t}\left(k\right),$$

and

(15) $$v_{i}^{t}\left(k\right)=z_{i}\left(k\right)-{\hat{z}}_{t}\left(k|k-1\right)$$

is the innovation of measurement for $z_{i}\left(k\right)\in Z_{t}\left(k\right)$. The filter gain is calculated as

(16) $$W_{t}\left(k\right)=P_{t}\left(k|k-1\right)H{\left(k\right)}^{\prime }S_{t}{\left(k\right)}^{-1}.$$

The covariance estimation for target *t* associated with the updated state is

(17) $$P_{t}\left(k|k\right)=\beta_{0}\left(k\right)P_{t}\left(k|k-1\right)+\left[1-\beta_{0}^{t}\left(k\right)\right]P_{t}^{c}\left(k|k\right)+{\tilde{P}}_{t}\left(k\right)$$

where the covariance of the state updated with the correct measurement, $P^{c}$, and the innovation spread, $\tilde{P}$, for target *t* are given by

(18) $$P_{t}^{c}\left(k|k\right)=P_{t}\left(k|k-1\right)-W_{t}\left(k\right)S_{t}\left(k\right)W_{t}{\left(k\right)}^{\prime }$$

and

(19) $${\tilde{P}}_{t}\left(k\right)=W_{t}\left(k\right)\left[\sum_{i=1}^{m_{t}\left(k\right)}\beta_{i}^{t}\left(k\right)v_{i}^{t}\left(k\right)v_{i}^{t}{\left(k\right)}^{\prime }-v_{t}\left(k\right)v_{t}{\left(k\right)}^{\prime }\right]W_{t}{\left(k\right)}^{\prime },$$

respectively.

The estimated state in PDAF from Equation (13) is for a single target $x_{t}$. To estimate the state of every target $x_{t}$, $t\in \{1,\ldots ,N_{T}\}$, we need to apply PDAF to the targets one by one sequentially. This means that for each target $x_{t}$, $t\in \{1,\ldots ,N_{T}\}$, we perform state and measurement prediction, form a validation region around the predicted measurement and prune off potentially unrelated measurements, obtain data-association probabilities for the validated measurements, and, finally, update the state estimate according to Equation (13). The order in which PDAF is applied to the targets is irrelevant because the outcome is independent of the order.

### 3.2. Distance-Weighting Probabilistic Data Association Filter

In the PDA algorithm, the association probability β is calculated as the likelihood ratio of a validated measurement to have originated from a target vs. from clutter. Chen et al. [4] have pointed out that a true measurement from a target of interest is more likely to be near the target’s predicted measurement. Since false alarms are uniformly distributed in the measurement space, the measurement nearest to that of the predicted measurement from a target of interest should carry more weight while calculating the association probabilities for the target. The distance weight proposed in [4] is calculated as

(20) $$\Delta_{i}^{t}\left(k\right)=\frac{1/\delta_{i}^{t}\left(k\right)}{\sum_{i=1}^{m_{t}\left(k\right)}1/\delta_{i}^{t}\left(k\right)}$$

where $\delta_{i}^{t}\left(k\right)$ is the Mahalanobis distance between validated measurement *i* and predicted measurement of target *t* at time *k*. The Mahalanobis distance is calculated as the norm of the innovation squared and is given by

(21) $$\delta_{i}^{t}\left(k\right)=v_{i}^{t}{\left(k\right)}^{\prime }S_{t}{\left(k\right)}^{-1}v_{i}^{t}\left(k\right)$$

where $v_{i}^{t}\left(k\right)$ is the innovation as defined in Equation (15).

The new data association probabilities for target *t* at time *k* are calculated as

(22) $$B_{i}^{t}\left(k\right)=\beta_{i}^{t}\left(k\right)\Delta_{i}^{t}\left(k\right)\;i=1,\ldots ,m_{t}\left(k\right)$$

and are normalized according to

(23) $$B_{i}^{t}\left(k\right)=B_{i}^{t}\left(k\right)/\sum_{j=0}^{m_{t}\left(k\right)}B_{i}^{t}\left(k\right)\;i=0,1,\ldots ,m_{t}\left(k\right).$$

The data association probabilities obtained in Equation (23) are used for estimating target state as explained previously in PDAF Section 3.1.4. Similar to PDAF, the DWPDA filter is designed for tracking a single target. For the purpose of tracking multiple targets, we need to update the states of the targets one by one.

### 3.3. Joint Probabilistic Data Association Filter

The PDA algorithm is designed for tracking a single target in clutter. Because the PDA algorithm assumes all the incorrect measurements in the validation region of a target of interest are clutter, it is susceptible to scenarios where these incorrect measurements might have originated from another nearby target. Situations may arise in MTT where the validation regions of nearby targets may overlap for several time frames in a row and cause persistent interference that can lead to track deterioration or track loss altogether with PDA. JPDA improves on PDA by calculating joint association probabilities at each time step. Marginalized association probabilities for each target can be obtained using these joint association probabilities, which are then used for estimating the state of each target.

#### 3.3.1. Measurement Validation

A major difference between the PDA and the JPDA algorithm is that no individual validation gates will be assumed for the various targets. A uniform validation region for all targets is obtained by taking a union of individual validation gates. This way each measurement is assumed to be validated for each target and false alarms will be assumed to be uniformly distributed across the entire validation region. Predictions for each target are done at each time step similar to the PDA algorithm using Equations (4)–(7).

Once the validated measurements are obtained for each target using Equations (8) and (10), the combined validated measurements at time *k* are given according to

(24) $$Z_{N_{T}}\left(k\right)=\overset{N_{T}}{\underset{i=1}{⋃}}Z_{i}\left(k\right).$$

The number of combined validated measurements is $m_{N_{T}}\left(k\right)$ at time *k*.

#### 3.3.2. The Validation Matrix

A validation matrix Ω of size $m_{N_{T}}\times \left(N_{T}+1\right)$ with binary elements is created using the combined validated measurement.

(25) $$\Omega \left(k\right)=\left[\omega_{jt}\right]\;j=1,\ldots ,m_{N_{T}}\left(k\right);\;t=0,1,\ldots ,N_{T}$$

where

(26) $$\omega_{jt}=\left\{\begin{array}{c} 1\;\mathrm{if}\;z_{j}\left(k\right)\in Z_{t}\left(k\right)\\ 0\;\mathrm{otherwise}. \end{array}\right.$$

The first column of Ω(k) corresponding to t=0 is all unity indicating that each measurement could be a false alarm.

#### 3.3.3. The Feasible Joint Events

The joint association events for time *k* are given by

(27) $$\theta \left(k\right)=\overset{m_{N_{T}}}{\underset{j=1}{⋂}}\theta_{jt_{j}}\;j=1,\ldots ,m_{N_{T}}\left(k\right);\;t=0,1,\ldots ,N_{T}$$

where in the event of $\theta_{jt}$, measurement *j* is originated from target $t_{j}$. An event matrix $\hat{\Omega }$ consisting of the units in Ω corresponding to the association in θ is used to represent a joint association event θ.

(28) $$\hat{\Omega }\left(\theta \left(k\right)\right)=\left[{\hat{w}}_{jt}\left(\theta \left(k\right)\right)\right]\;j=1,\ldots ,m_{N_{T}}\left(k\right);\;t=0,1,\ldots ,N_{T}$$

where

(29) $${\hat{\omega }}_{jt}\left(\theta \left(k\right)\right)=\left\{\begin{array}{c} 1\;\mathrm{if}\;\theta_{jt}\in \theta \left(k\right)\\ 0\;\mathrm{otherwise}. \end{array}\right.$$

Not all joint association events are feasible joint events. As per our target tracking model assumptions, feasible association events are only those where no more than one measurement is associated with each target. Therefore a feasible association event is the one which satisfies the following two conditions:

1. A measurement can have only one source, i.e.,
  (30) $$\sum_{t=0}^{N_{T}}{\hat{\omega }}_{jt}\left(\theta \left(k\right)\right)=1\;\forall j.$$
2. Each target can generate at most one measurement, i.e.,
  (31) $$\delta_{t}\left(\theta \left(k\right)\right)\overset{\Delta }{=}\sum_{j=1}^{m_{N_{T}}}{\hat{\omega }}_{jt}\left(\theta \left(k\right)\right)\leq 1\;t=1,\ldots ,N_{T}.$$
  $\delta_{t}\left(\theta \left(k\right)\right)$ in Equation (31), called the target detection indicator, indicates if a measurement is associated with target *t* in event θ(k). Similarly, we define the measurement association indicator τ(θ(k)) to indicate if measurement *j* is associated with a target in event θ(k).
  (32) $$\tau_{j}\left(\theta \left(k\right)\right)\overset{\Delta }{=}\sum_{t=1}^{N_{T}}{\hat{\omega }}_{jt}\left(\theta \left(k\right)\right)\;j=1,\ldots ,m_{N_{T}}\left(k\right).$$

We can obtain the number of false alarms in event θ(k) as

(33) $$\phi \left(\theta \left(k\right)\right)=\sum_{j=1}^{m_{N_{T}}\left(k\right)}\left[1-\tau_{j}\left(\theta \left(k\right)\right)\right].$$

#### 3.3.4. Joint Data Association Probabilities

The joint data association probabilities are calculated as

(34) $$P\left\{\theta \left(k\right)|Z^{k}\right\}=\frac{1}{c}\left[Z_{m_{N_{T}}\left(k\right)}\left(k\right)|\theta \left(k\right),m_{N_{T}}\left(k\right),Z^{k-1}\right]P\left\{\theta \left(k\right)|m_{N_{T}}\left(k\right)\right\}$$

where P{·} indicates the probability mass function (PMF) and *c* is the normalization constant. The marginal association probabilities are obtained from the joint DA probabilities by summing over all the joint events according to Equation (29). The marginal association probabilities at time *k* are then given as

(35) $$\begin{array}{cc} \beta_{j}^{t}\left(k\right) & =\sum_{\theta (k)}P\left\{\theta \left(k\right)|Z^{k}\right\}{\hat{\omega }}_{jt}\left(\theta \left(k\right)\right)\;j=1,\ldots ,m_{N_{T}}\left(k\right);\;t=0,1,\ldots ,N_{T} \end{array}$$

(36) $$\begin{array}{cc} \beta_{0}^{t}\left(k\right) & =1-\sum_{j=1}^{m_{N_{T}}\left(k\right)}\beta_{j}^{t}\left(k\right)\;t=0,1,\ldots ,N_{T} \end{array}$$

where the probability $\beta_{j}^{t}$ represents that the measurement *j* is associated with target *t* and $\beta_{0}^{t}$ indicates that none of the validated measurements is associated with target *t*. The expression for the joint association probabilities in Equation (35) can be given in terms of the variables defined in JPDAF Section 3.3.3 as

(37) $$P\left\{\theta \left(k\right)|Z^{k}\right\}=\frac{1}{c_{1}}\prod_{j=1}^{m_{N_{T}}\left(k\right)}{\left\{\lambda^{-1}\left[\Lambda_{j}^{t_{j}}\left(k\right)\right]\right\}}^{\tau_{j}\left(\theta \left(k\right)\right)}\prod_{t=1}^{N_{T}}{\left(P_{D}\right)}^{\delta_{t}\left(\theta \left(k\right)\right)}{\left(1-P_{D}\right)}^{1-\delta_{t}\left(\theta \left(k\right)\right)}$$

where $\Lambda_{j}^{t_{j}}$ is the Gaussian density of measurement *j* associated with target of index $t_{j}$ given by

(38) $$\Lambda_{j}^{t_{j}}\left(k\right)=N\left[z_{j}\left(k\right);{\hat{z}}_{t_{j}}\left(k|k-1\right),S_{t_{j}}\left(k\right)\right]$$

and $c_{1}$ is the new normalization constant. The marginal association probabilities obtained in Equations (35) and (36) are used in conjunction with the state estimation equations given in PDAF Section 3.1.4 for estimating state of each target separately.

### 3.4. Loopy Sum-Product Algorithm

Graphical models can be used for representing the joint probability distributions of many variables efficiently by exploiting factorization. For SPA, factor graphs are utilized for representing the DA relations between multiple targets and their validated measurements. SPA is then conducted on the resulting loopy factor graph for obtaining the marginal DA probabilities. The algorithm for the loopy-SPA can be given as follows.

#### 3.4.1. Belief Propagation in Factor Graphs

Kschischang et al. [3] demonstrated how factor graphs can be used to interpret a variety of algorithms such as the KF, the Viterbi algorithm, the Hidden Markov Model, etc. A factor graph is a standard bipartite graphical representation of a mathematical relationship between random variables and local functions. While formulating a factor graph to express the structure of the factorization of a global function of many variables into several local functions, each node represents each random variable n∈N, each factor represents each local function f∈F, and an edge connects a node *n* to a factor *f* if and only if *n* is an argument of *f*.

The SPA algorithm, also called as Belief Propagation (BP), passes messages, called beliefs, between nodes and factors in an iterative manner for conducting optimal inference on a tree-structured factor graph. We denote by $\psi_{\cdot }\left(\cdot \right)$ the joint probability distribution function, by $\mu_{n\to f}\left(x_{n}\right)$ the message sent from node $n\in \eta_{f}$ to factor *f*, by $\mu_{f\to n}\left(x_{n}\right)$ the message sent from factor *f* to node $n\in \eta_{f}$, by $\eta_{f}\subseteq N$ the set of neighboring nodes of *f*, and by $\eta_{n}=\left\{f\in F|n\in \eta_{f}\right\}$ the factors involving node *n*. The message computation performed by SPA can be given as

(39) $$\begin{array}{cc} \mu_{n\to f}\left(x_{n}\right) & =\prod_{\xi \in \eta_{n}\backslash \left\{f\right\}}\mu_{\xi \to n}\left(x_{n}\right) \end{array}$$

(40) $$\begin{array}{cc} \mu_{f\to n}\left(x_{n}\right) & =\sum_{∼\{x_{n}\}}\left(\psi_{f}\left(x_{\eta_{f}}\right)\prod_{\xi \in \eta_{f}\backslash \left\{n\right\}}\mu_{\xi \to f}\left(x_{\xi }\right)\right) \end{array}$$

where $∼\{x_{n}\}$ denotes the summation over all arguments of *f* except $x_{n}$. For factorization involving continuous variables, the summation is replaced with an integral taken over a Lebesgue measure. The algorithm is known as sum-product because the steps involved are summations (or integrals) and products of factors and messages.

SPA extended to loopy graphs is called LSPA. LSPA simply requires repeated application of SPA until convergence occurs. Practically, this means computing messages continuously using Equations (39) and (40) until the maximum error between subsequent messages is less than a pre-set threshold. However, LSPA is neither guaranteed to converge to the right answer nor to converge at all.

#### 3.4.2. Factor Graphs for Data Association

We consider the DA problem involving known and fixed $N_{T}$ targets and their combined validated measurements $Z_{N_{T}}\left(k\right)$ at time *k* obtained as explained in JPDAF Section 3.3.1. The number of combined validated measurements are $m_{N_{T}}\left(k\right)$ at time *k*. Formulating a factor graph for the DA problem that is guaranteed to converge according to [5,14,15], we need the following two sets of association variables:

1. Target oriented association variable (*a*): Create an association variable $a_{i}\left(k\right)\in \{0,1,\ldots ,$$m_{N_{T}}\left(k\right)\}$ for each target $i\in \{1,\ldots ,N_{T}\}$. The value assigned to $a_{i}\left(k\right)$ is an index to the measurement with which target *i* is hypothesized to be associated at time *k* (zero if the target is hypothesized to not have been detected). The complete set of target oriented association variables at time *k* is denoted by a(k).
2. Measurement oriented association variable (*b*): Create an association variable $b_{j}\left(k\right)\in \left\{0,1,\ldots ,N_{T}\right\}$ for each measurement $j\in \{1,\ldots ,m_{N_{T}}\left(k\right)\}$. The value assigned to $b_{j}\left(k\right)$ is an index to the target with which measurement *j* is hypothesized to be associated at time *k* (zero if the measurement is hypothesized to be clutter). The complete set of measurement oriented association variables at time *k* is denoted by b(k).

**Figure 1:**
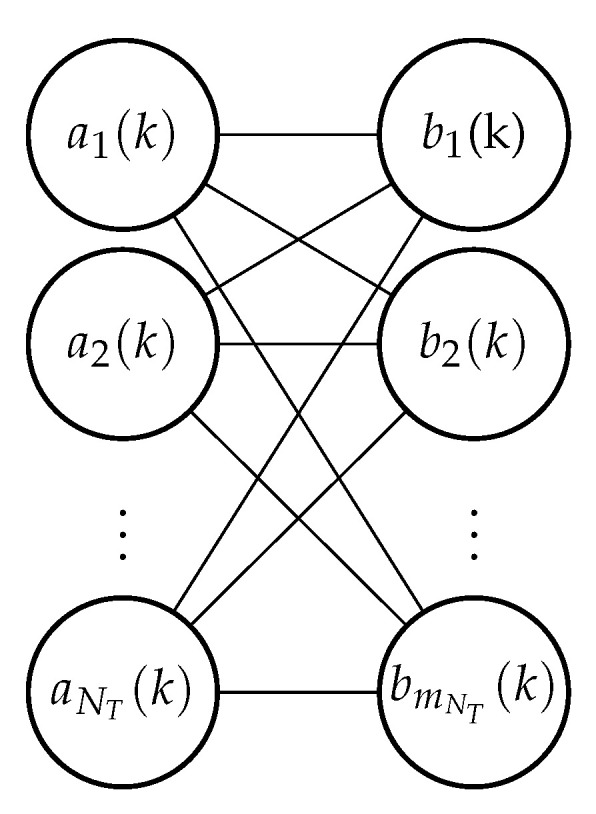
Bipartite graphical model formulation for data association at time *k*. The value assigned to $a_{i}\left(k\right)$ is an index to the measurement with which target *i* is hypothesized to be associated at time *k* and the value assigned to $b_{j}\left(k\right)$ is an index to the target with which measurement *j* is hypothesized to be associated at time *k*.

Given one set of association variables, the other set can be perfectly reconstructed. As shown in [5,14,15], this use of seemingly redundant information while forming a factor graph leads to the remarkable result of guaranteed convergence of LSPA computed on the said factor graph. A bipartite graphical model formed using the association variables a(k) an b(k) is shown in Figure 1.

Our goal is to estimate state vectors $x_{i}\left(k\right)$, $i\in \{1,\ldots ,N_{T}\}$ from the measurement vectors Z(k) at time *k*. Introducing the data-association variables a(k) and b(k), a Bayesian approach to state estimation of $x_{i}\left(k\right)$ produces a posterior probability density function (PDF) $f(x_{i}\left(k\right),a\left(k\right),b\left(k\right)|Z\left(k\right))$. This can be achieved by marginalizing the joint posterior PDF f(X(k),a(k),b(k)|Z(k)), where $X\left(k\right)=(x_{i}\left(k\right),\ldots ,x_{N_{T}}\left(k\right))$. However, direct marginalization of f(X(k),a(k),b(k)|Z(k)) is not always feasible. Assuming that the posterior PDF of X(k) factorizes into a product of terms associated with each target $x_{i}\left(k\right)$, an efficient marginalization is given as

(41) $$f\left(X\left(k\right),a\left(k\right),b\left(k\right)\right)\propto \prod_{i=1}^{N_{T}}\psi_{i}\left(x_{i}\left(k\right),a_{i}\left(k\right)\right)\prod_{j=1}^{m_{N_{T}}\left(k\right)}\psi_{i,j}\left(a_{i}\left(k\right),b_{j}\left(k\right)\right)$$

where

(42) $$2\psi_{i}\left(x_{i}\left(k\right),a_{i}\left(k\right)\right)=\left\{\begin{array}{cc} {}[1-P_{D}\left(x_{i}\left(k\right)\right)f\left(x_{i}\left(k\right)|Z\left(k-1\right)\right)]\; & \mathrm{if}\;a_{i}\left(k\right)=0\\ {}_{D}\left(x_{i}\left(k\right)\right)f\left(z_{a_{i}\left(k\right)}|x_{i}\left(k\right)\right)f\left(x_{i}\left(k\right)|Z\left(k-1\right)\right)]/\lambda \left(z_{a_{i}\left(k\right)}\right)\; & \mathrm{if}\;a_{i}\left(k\right)\neq 0 \end{array}\right.$$

and

(43) $$\psi_{i,j}\left(a_{i}\left(k\right),b_{j}\left(k\right)\right)=\left\{\begin{array}{cc} 0\; & \mathrm{if}\;a_{i}\left(k\right)=j,\;b_{j}\left(k\right)\neq i\;\mathrm{or}\;b_{j}\left(k\right)=i,\;a_{i}\left(k\right)\neq j\;\;\;\;\;\;\;\;\\ 1\; & \mathrm{otherwise}. \end{array}\right.$$

Here, ∝ indicates equality up to a normalization factor, $\psi_{i}\left(x_{i}\left(k\right),a_{i}\left(k\right)\right)$ indicates the dependence of the factors $\psi_{i}\left(\cdot \right)$ on Z(k), $\psi_{i,j}\left(a_{i}\left(k\right),b_{j}\left(k\right)\right)$ enforces consistency of the redundant association variables $a_{i}\left(k\right)$ and $b_{j}\left(k\right)$ describing the same association configuration, $P_{D}\left(x_{i}\right)$ gives the probability of detection for target $x_{i}$, and $\lambda (z_{j})$ gives the PDF value of measurement $z_{j}$ occurring as a result of false alarm with a Poisson point process.

**Figure 2:**
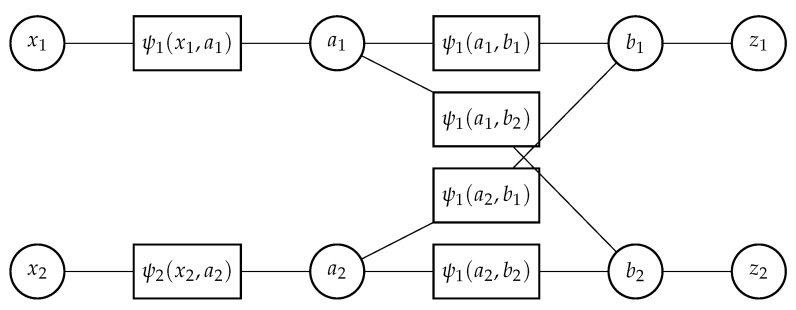
Factor graph representing the factorization of the joint posterior probability density function (PDF) $f(x_{1},x_{2},a_{1},a_{2},b_{1},b_{2}|z_{1},z_{2})$ according to Equation (41), depicted for one time step. For simplicity, the time index *k* is omitted.

The factorization structure in Equation (41) can be represented by a factor graph. As an example, for $X\left(k\right)=(x_{1}\left(k\right),x_{2}\left(k\right))$ and $Z\left(k\right)=(z_{1}\left(k\right),z_{2}\left(k\right))$, the factor-graph representation of Equation (41) is shown in Figure 2. Since the target states $x_{i}\left(k\right)$ in Figure 2 are leaf nodes, we can marginalize these and replace the factor $\psi_{i}\left(x_{i}\left(k\right),a_{i}\left(k\right)\right)$ with $\psi_{i}\left(a_{i}\left(k\right)\right)$. In a factor graph, each parameter variable is represented by a variable node (circle node), and each factor is represented by a rectangle node, as shown in Figure 2. Each variable node and each factor node are connected by an edge if the variable is an argument of the factor. For each node, certain messages are calculated according to Equations (39) and (40). Message passing is started at variable nodes with only one edge (which pass a constant message) and/or factor nodes with only one edge (which pass the corresponding factor). Finally, for each variable node, a belief (posterior PDF value) is calculated as the product of all incoming messages (passed from all adjacent factor nodes) followed by a normalization. For a tree-structured factor graph, these beliefs are exactly equal to marginal posterior PDF values. For a loopy factor graph, the beliefs are in general only approximations of the respective marginal posterior PDF values. A detailed description of the messages passed between each variable node and factor node is given in [19]. A sample MATLAB program for implementing one iteration of loopy SPA is given in [14].

#### 3.4.3. Joint Data Association Probabilities

Once the combined validated measurements are obtained as given in Equation (24), the joint data association probabilities under the stated assumptions at time *k* are calculated as

(44) $$p\left(a\left(k\right),b\left(k\right)|Z^{k}\right)=\prod_{i=1}^{N_{T}}\psi_{i}\left(a_{i}\left(k\right)\right)\prod_{j=1}^{m_{N_{T}}\left(k\right)}\psi_{i,j}\left(a_{i}\left(k\right),b_{j}\left(k\right)\right)$$

where

(45) $$\psi_{i,j}\left(a_{i}\left(k\right),b_{j}\left(k\right)\right)=\left\{\begin{array}{c} 0\;\mathrm{if}\;a_{i}\left(k\right)=j,\;b_{j}\left(k\right)\neq i\;\mathrm{or}\;b_{j}\left(k\right)=i,\;a_{i}\left(k\right)\neq j\\ 1\;\mathrm{otherwise} \end{array}\right.$$

enforce consistency of the redundant association variables $a_{i}\left(k\right)$ and $b_{j}\left(k\right)$ describing the same association configuration, and $\psi_{i}\left(a_{i}\left(k\right)=0\right)=1$ and $\psi_{i}\left(a_{i}\left(k\right)=j>0\right)$ are the (unnormalized) single target data association probabilities obtained as explained in PDAF Section 3.1.3. Equation (44) can be expressed as the formulation of SPA for a bipartite model illustrated in Figure 1 in which all target association variables $a_{i}\left(k\right)$ are connected to all measurement association variables $b_{j}\left(k\right)$. In this case, LSPA may be implemented via two half iterations, alternating between the two sets of messages $\mu_{a_{i}\left(k\right)\to b_{j}\left(k\right)}\left(b_{j}\left(j\right)\right)$ and $\mu_{b_{j}\left(j\right)\to a_{i}\left(k\right)}a_{i}\left(k\right)$. The message updating equations according to Equations (39) and (40) can be given as

(46) $$\begin{array}{cc} \mu_{a_{i}\left(k\right)\to b_{j}\left(k\right)}\left(b_{j}\left(k\right)\right) & =\sum_{a_{i}\left(k\right)}\psi_{i}\left(a_{i}\left(k\right)\right)\psi_{i,j}\left(a_{i}\left(k\right),b_{j}\left(k\right)\right)\prod_{j^{\prime }\neq j}\mu_{b_{j^{\prime }\left(k\right)}\to a_{i}\left(k\right)}\left(a_{i}\left(k\right)\right) \end{array}$$

(47) $$\begin{array}{cc} & =\left\{\begin{array}{cc} \psi_{i}\left(j\right)\prod_{j^{\prime }\neq j}\mu_{b_{j^{\prime }\left(k\right)}\to a_{i}\left(k\right)}\left(j\right)\; & \mathrm{if}\;b_{j}\left(k\right)=i\\ \sum_{a_{i}\left(k\right)\neq j}\psi_{i}\left(a_{i}\left(k\right)\right)\prod_{j^{\prime }\neq j}\mu_{b_{j^{\prime }}\left(k\right)\to a_{i}\left(k\right)}\left(a_{i}\left(k\right)\right)\; & \mathrm{if}\;b_{j}\left(k\right)\neq i \end{array}\right. \end{array}$$

and

(48) $$\begin{array}{cc} \mu_{b_{j}\left(k\right)\to a_{i}\left(k\right)}\left(a_{i}\left(k\right)\right) & =\sum_{b_{j}\left(k\right)}\psi_{i,j}\left(a_{i}\left(k\right),b_{j}\left(k\right)\right)\prod_{i^{\prime }\neq i}\mu_{a_{i^{\prime }}\left(k\right)\to b_{j}\left(k\right)}\left(b_{j}\left(k\right)\right) \end{array}$$

(49) =∏i′≠iμai′→j(i)ψi(ai(k))∏j′≠jai(k)(ai(k))ifai(k)=j∑bj(k)≠i∏i′≠iμai′→j(bj(k))ifai(k)≠j.

These messages can be further simplified as shown in [14,15]. Upon convergence of LSPA, the approximate marginal association probabilities can be given as

(50) $$\begin{array}{cc} p(a_{i}\left(k\right)=j|Z^{k}) & =\frac{\psi_{i}\left(j\right)\mu_{b_{j}\left(k\right)\to a_{i}\left(k\right)}\left(a_{i}\left(k\right)\right)}{\sum_{j^{\prime }=0}^{m_{N_{T}}\left(k\right)}\psi_{i}\left(j^{\prime }\right)\mu_{b_{j^{\prime }}\left(k\right)\to a_{i}\left(k\right)}\left(a_{i}\left(k\right)\right)} \end{array}$$

(51) $$\begin{array}{cc} p(b_{j}\left(k\right)=i|Z^{k}) & =\frac{\mu_{a_{i}\left(k\right)\to b_{j}\left(k\right)}\left(b_{j}\left(k\right)\right)}{\sum_{i^{\prime }=0}^{N_{T}}\mu_{a_{i^{\prime }}\left(k\right)\to b_{j}\left(k\right)}\left(b_{j}\left(k\right)\right)} \end{array}$$

where $\mu_{b_{j}\left(k\right)=0\to a_{i}\left(k\right)}\left(a_{i}\left(k\right)\right)\overset{\Delta }{=}1$ and $\mu_{a_{i}\left(k\right)=0\to b_{j}\left(k\right)}\left(b_{j}\left(k\right)\right)\overset{\Delta }{=}1$. The marginal association probabilities are used to estimate target states as per PDAF Section 3.1.4.

### 3.5. Distance-Weighting Loopy Sum-Product Algorithm

Since the factor-graph representation for the MTT DA problem contains loops, and the convergence process of calculating the marginal data-association probabilities using LSPA is governed by some heuristically determined preset threshold, different initiation messages can lead to different final beliefs. These initiation messages for the convergence process happen to be the (unnormalized) single-target data-association probabilities. These probabilities directly influence the marginal data-association probabilities at the end of the convergence process and, consequently, the estimation of the target state. We would expect that a more accurate initial set of single-target probabilities leads to either more accurate final beliefs, a faster convergence to the final beliefs, or both. The distance-weight-based association probabilities have been proven to be more accurate for tracking a single target in densely cluttered environments [4]. We would expect this adjustment to the initial condition to change the calculation of the association probabilities and hence the overall tracking process in terms of tracking accuracy or computation time, and therefore worth exploring.

Here we formulate a modification for the joint data association probabilities calculated using LSPA under the stated assumptions as described in Section 3.4. The distance-weight based joint data association probabilities at time *k* can be given as

(52) $$p\left(a\left(k\right),b\left(k\right)|Z^{k}\right)=\prod_{i=1}^{N_{T}}\psi_{i}\left(a_{i}\left(k\right)\right)\prod_{j=1}^{m_{N_{T}}\left(k\right)}\psi_{i,j}\left(a_{i}\left(k\right),b_{j}\left(k\right)\right)$$

where

(53) $$\psi_{i,j}\left(a_{i}\left(k\right),b_{j}\left(k\right)\right)=\left\{\begin{array}{c} 0\;a_{i}\left(k\right)=j,\;b_{j}\left(k\right)\neq i\;\mathrm{or}\;b_{j}\left(k\right)=i,\;a_{i}\left(k\right)\neq j\\ 1\;\mathrm{otherwise} \end{array}\right.$$

enforce consistency of the redundant association variables $a_{i}\left(k\right)$ and $b_{j}\left(k\right)$ describing the same association configuration, and $\psi_{i}\left(a_{i}\left(k\right)=0\right)=1$ and $\psi_{i}\left(a_{i}\left(k\right)=j>0\right)=B_{i}^{t}\left(k\right)$ are the (unnormalized) distance-weight based single target data association probabilities obtained in Equation (22). The approximated marginal association probabilities are obtained from the joint DA probabilities as described in Section 3.4.

## 4. Simulation and Analysis

### 4.1. The Dynamic Model

We assume a two-dimensional system where the target state vector consists of position and velocity in each of the two coordinates. For target *i* at time *k*, the target state $x_{i}\left(k\right)=\left[x\left(k\right),\dot{x}\left(k\right),y\left(k\right),\dot{y}\left(k\right)\right]$ has four components: The first and second components are the horizontal location and velocity, respectively, while the third and fourth components are the vertical location and velocity, respectively. The system is equipped with the nearly constant velocity (NCV) model (also sometimes called the constant velocity (CV) model). The system is described by Equations (1) and (2) with

(54) $$\begin{array}{cc} F(k) & =\left[\begin{array}{cccc} 1 & T & 0 & 0\\ 0 & 1 & 0 & 0\\ 0 & 0 & 1 & T\\ 0 & 0 & 0 & 1 \end{array}\right] \end{array}$$

(55) $$\begin{array}{cc} G(k) & =\left[\begin{array}{cc} T^{2}/2 & 0\\ T & 0\\ 0 & T^{2}/2\\ 0 & T \end{array}\right] \end{array}$$

(56) $$\begin{array}{cc} H(k) & =\left[\begin{array}{cccc} 1 & 0 & 0 & 0\\ 0 & 0 & 1 & 0 \end{array}\right] \end{array}$$

(57) $$\begin{array}{cc} Q(k) & =\left[\begin{array}{cc} q & 0\\ 0 & q \end{array}\right] \end{array}$$

(58) $$\begin{array}{cc} R(k) & =\left[\begin{array}{cc} r & 0\\ 0 & r \end{array}\right] \end{array}$$

where *T* is the sampling interval.

### 4.2. Simulation Parameters

For our simulations, we set the probability of detection $P_{D}=0.9$, the gate threshold γ=9.21 corresponding to gate probability $P_{G}=0.99$, sampling interval T=1 s, the process noise variance q=0.05, and the measurement noise variance r=5. To better evaluate the accuracy of different algorithms under multiple conditions, we varied the clutter density generated according a Poisson point process from $\lambda =1.0\times 10^{-4}/\mathrm{scan}/m^{2}$ to $\lambda =5.0\times 10^{-4}/\mathrm{scan}/m^{2}$. We also varied the number of tracked targets from 1 to 6. The initial state of the first target is always set at $x_{1}\left(1\right)=\left[100\;m,\;30\;m/s,\;100\;m,\;30\;m/s\right]$. We set the initial state of each consecutive target by $x_{i}\left(1\right)=\left[100\;m,\;30\;m/s,\;\left(100-i\times 100\times c\left(i\right)\right)\;m,\;\left(30-i\times 30\times c\left(i\right)\right)\;m/s\right]$ where *i* is the target index and c(i) is a single uniformly distributed random number in the interval (0,1) independent of each other. To compare the performance, we performed 500 Monte Carlo simulations on MATLAB (Natick, MA, USA) [21].

### 4.3. Results and Discussion

When choosing an optimal tracking algorithm, there is typically a trade off between tracking accuracy and computation time. Maintaining a high-level accuracy in complex scenarios where multiple targets need to be tracked simultaneously and the environment is particularly noisy requires significant computation time. Traditionally, the tracking accuracy of an estimator is calculated in terms of a miss-distance, or localization error, between a reference value and its estimated value. In our context, we are also interested in evaluating missed detections and false alarms. The generalized optimal sub-pattern assignment (GOSPA) metric has been designed to reflect this performance [22]. Informally, the GOSPA metric can be defined as

$$\mathrm{GOSPA}=\sum \mathrm{localization}\;\mathrm{error}+\frac{\mathrm{cutoff}\;\mathrm{distance}}{2}\left(\#\;\mathrm{of}\;\mathrm{missed}\;\mathrm{detections}+\#\;\mathrm{of}\;\mathrm{false}\;\mathrm{alarms}\right)$$

(for a precise detailed description of GOSPA, see [22]). The localization error is for pairs of true targets and target estimates that are sufficiently close. A missed detection is declared if there is no corresponding target sufficiently close to it, and a false alarm is declared if there is no corresponding true target sufficiently close to it. To evaluate the performance of the different tracking algorithms described in Section 3 in terms of the miss-distance, the GOSPA metric, and computation time, we considered multiple scenarios with varying levels of complexities.

**Figure 3:**
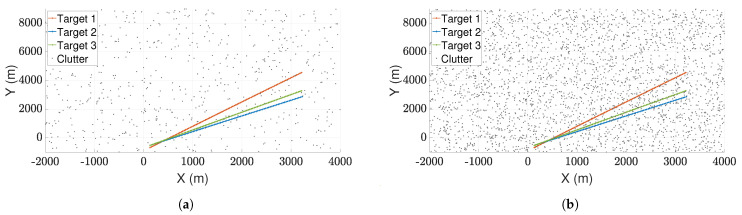
True target positions for three crossing targets with different clutter densities. (**a**) Clutter density $\lambda =1\times 10^{-4}/$m${}^{2}$; and (**b**) clutter density $\lambda =5\times 10^{-4}/$m${}^{2}$.

**Figure 4:**
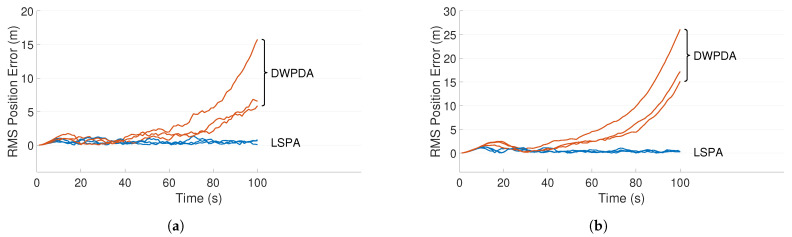
RMS position error for three crossing targets using DWPDA and LSPA with different clutter densities. (**a**) Clutter density $\lambda =1\times 10^{-4}/$m${}^{2}$; and (**b**) clutter density $\lambda =5\times 10^{-4}/$m${}^{2}$.

**Figure 5:**
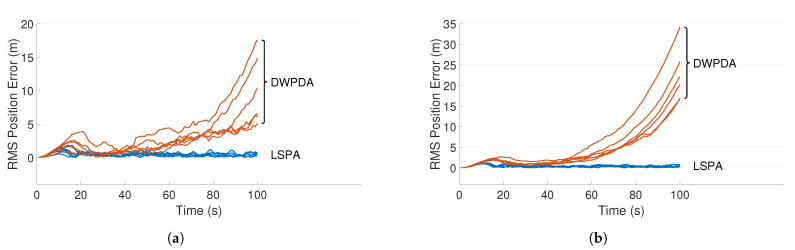
RMS position error for six crossing targets using DWPDA and LSPA with different clutter densities. (**a**) Clutter density $\lambda =1\times 10^{-4}/$m${}^{2}$; and (**b**) clutter density $\lambda =5\times 10^{-4}/$m${}^{2}$.

**Figure 6:**
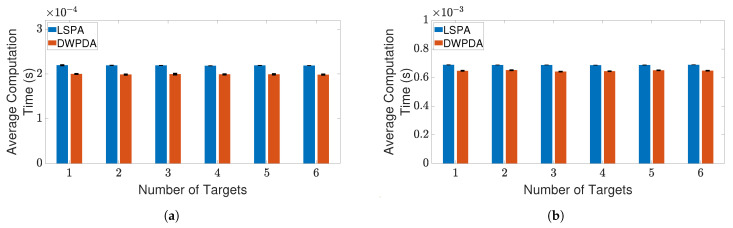
Average computation time for obtaining association probabilities using DWPDA and LSPA for tracking multiple crossing targets with different clutter densities. (**a**) Clutter density $\lambda =1\times 10^{-4}/$m${}^{2}$; and (**b**) clutter density $\lambda =5\times 10^{-4}/$m${}^{2}$. Error bars indicate 95% confidence intervals.

Figure 3a,b depict two such scenarios where the trajectories of three targets are shown for clutter densities $\lambda =1\times 10^{-4}/$m${}^{2}$ and $\lambda =5\times 10^{-4}/$m${}^{2}$, respectively. The scenario depicted in Figure 3b is more demanding because of the increased number of false alarms. Figure 4a,b compares the performance of LSPA and DWPDA in terms of tracking accuracy, calculated using the root mean square (RMS) position error, over a period of 100-time intervals for the above two scenarios, respectively. We see that the RMS position errors for LSPA stay close to 0 over the entire period while they are always increasing for DWPDA as time progresses. We see similar results while tracking six crossing targets with clutter densities $\lambda =1\times 10^{-4}/$m${}^{2}$ and $\lambda =5\times 10^{-4}/$m${}^{2}$, as depicted in Figure 5a,b. Figure 6a,b show that the computation times for the two clutter scenarios as the number of targets increases from one to six. We see that the computation times required for LSPA are only slightly higher than those of DWPDA. Finally, Table 1 and Table 2 summarize the results for the performance in terms of the GOSPA metric based on the Euclidean distance with a cutoff parameter of 30. Table 1 shows the average GOSPA errors as we increase the number of targets from 1 to 6 while keeping the clutter density constant at $\lambda =3\times 10^{-4}/$m${}^{2}$. We can see that irrespective of the number of targets that are being tracked, GOSPA errors for LSPA are only a fraction of the errors for DWPDA. We see a similar pattern in Table 2 where we increase the clutter density from $\lambda =1\times 10^{-4}/$m${}^{2}$ to $\lambda =5\times 10^{-4}/$m${}^{2}$ while keeping the number of targets fixed. The poor performance of DWPDA is expected, since the algorithm is ill-equipped to deal with the problem of DA in MTT, and GOSPA appropriately penalizes any missed detections and false alarms. From the above results, it is evident that LSPA is superior to DWPDA in terms of tracking accuracy in all scenarios without trading off much computation time. This superior tracking accuracy can be attributed to the reduction in the association probabilities of false measurements in the overlapping validation regions from multiple targets and, at the same time, the increase in the association probabilities for actual target measurements. From the above results, we know that LSPA can improve the tracking performance for MTT in densely cluttered environments.

**Figure 7:**
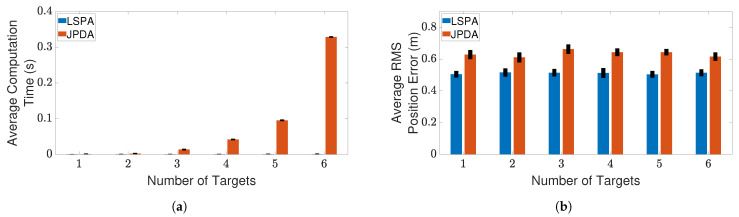
Tracking multiple crossing targets using LSPA and JPDA with clutter density $\lambda =1\times 10^{-4}/$m${}^{2}$. (**a**) Average computation time for obtaining association probabilities; (**b**) average RMS position error. Error bars indicate 95% confidence intervals.

**Figure 8:**
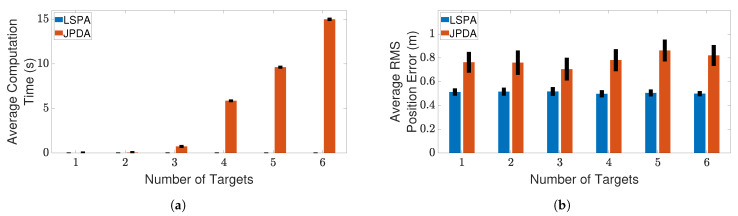
Tracking multiple crossing targets using loopy sum-product algorithm (LSPA) and joint probabilistic data association (JPDA) with clutter density $\lambda =5\times 10^{-4}/$m${}^{2}$. (**a**) Average computation time for obtaining association probabilities; (**b**) average root mean square (RMS) position error. Error bars indicate 95% confidence intervals.

**Figure 9:**
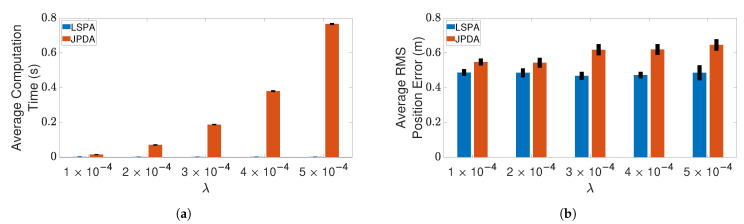
Tracking three crossing targets using LSPA and JPDA. (**a**) Average computation time for obtaining association probabilities; (**b**) average RMS position error. Error bars indicate 95% confidence intervals.

Next, we compare the performance of LSPA with JPDA, in terms of computation time and RMS position error, as the number of targets increases from one to six. Figure 7 and Figure 8 show the results of this comparison for the two clutter scenarios. We can clearly see that the average computation time of LSPA is much smaller than that of JPDA, so much so that the LSPA computation times are not even visible in Figure 7a and Figure 8a. However, in contrast to the comparison of LSPA and DWPDA in terms of the RMS position error, in the case of LSPA compared with JPDA, Figure 7b and Figure 8b clearly show that there is little difference in RMS position error. The same observation applies in terms of computation time and RMS position error in Figure 9a,b respectively when we compare LSPA with JPDA in scenarios involving a fixed number of targets as the clutter density increases from $\lambda =1\times 10^{-4}/$m${}^{2}$ to $\lambda =5\times 10^{-4}/$m${}^{2}$. While there is little difference between LSPA and JPDA in terms of RMS position errors, Table 1 and Table 2 show that GOSPA errors for LSPA are less than 1/3rd of GOSPA errors for JPDA across almost all scenarios. These relatively higher GOSPA errors for JPDA can be explained by a few missed detections when multiple target paths overlap. These missed detections are rightly penalized in the GOSPA metric. These results show that LSPA dominates JPDA in terms of computation time while maintaining a high level of tracking accuracy. This dramatic reduction in computation time for LSPA can be explained by the implementation of the loopy factor graph, resulting in simultaneous updating of the joint association probabilities for multiple targets during each iteration of LSPA. The results show that LSPA scales well for real-time applications involving complex tracking scenarios.

**Figure 10:**
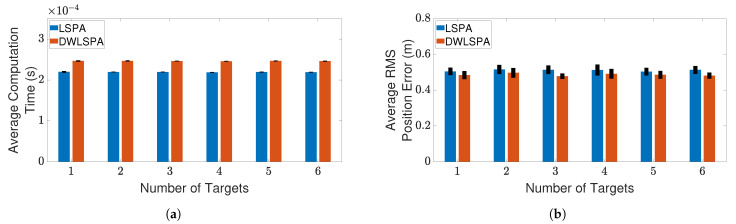
Tracking multiple crossing targets using LSPA and DWLSPA with clutter density $\lambda =1\times 10^{-4}/$m${}^{2}$. (**a**) Average computation time for obtaining association probabilities; (**b**) average RMS position error. Error bars indicate 95% confidence intervals.

**Figure 11:**
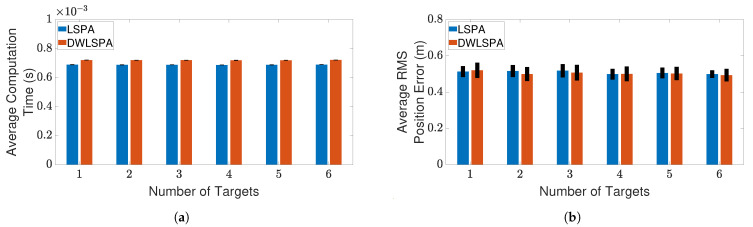
Tracking multiple crossing targets using LSPA and DWLSPA with clutter density $\lambda =1\times 10^{-4}/$m${}^{2}$.(**a**) Average computation time for obtaining association probabilities; (**b**) average RMS position error. Error bars indicate 95% confidence intervals.

**Figure 12:**
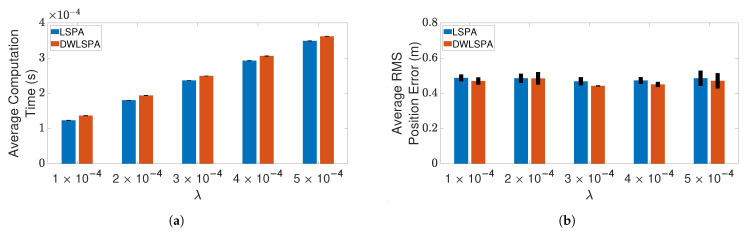
Tracking three crossing targets using LSPA and DWLSPA. (**a**) Average computation time for obtaining association probabilities; (**b**) average RMS position error. Error bars indicate 95% confidence intervals.

Finally, to see whether the integration of LSPA with the distance-weighting scheme has any effect on its performance, we compare LSPA with DWLSPA in terms of the same two metrics as above. Figure 10 and Figure 11 show the results of this comparison for two clutter scenarios respectively, as the number of targets increases from one to six. There is little difference between LSPA and DWLSPA in terms of computation times, as shown in Figure 10a and Figure 11a. Similarly, we can see that the RMS position errors for LSPA and DWLSPA are almost identical in Figure 10b and Figure 11b. Figure 12a,b show that the difference between LSPA and DWLSPA remains negligible, in terms of both computation time and RMS position error, for scenarios with a fixed number of targets and varying clutter densities. Table 1 and Table 2 show that GOSPA errors for LSPA and DWLSPA are comparable across all tested scenarios. This means that in addition to the localization errors, the missed detections and false alarms remain consistent between LSPA and DWLSPA, and the potential advantage of DWLSPA with the additional distance-weighting information is not apparent. Surprisingly, LSPA and DWLSPA perform equally well in every scenario in terms of both tracking accuracy and computation time. The unchanged performance of DWLSPA can be explained by the initiation of LSPA with small improvements in single-target association probabilities having insignificant effect on joint association probabilities calculated at the end of a large number of iterations. However, this unexpected lack of improvement contrasts sharply with results reported in [4] showing significant improvement when introducing distance weighting relative to PDA. This result is interesting and useful because we can see that distance weighting does not always lead to better performance. Moreover, the following important observation remains: LSPA is reliable and efficient for tracking multiple objects in cluttered environments.

## 5. Conclusions

In this paper, we formulated a distance-weighting PDA approach for LSPA and examined its effect for tracking multiple objects in cluttered environments. It has been previously shown that a modification of PDA according to a weighting scheme based on distances between predicted and true target positions improves the tracking accuracy of PDA. LSPA is known to be better than PDA and JPDA and, since PDA constitutes a crucial building block of LSPA, we expected the integration of DWPDA with LSPA to boost the overall performance even further. We studied the performance of LSPA against DWPDA, JPDA, and DWLSPA for a wide range of tracking scenarios involving multiple targets and varying clutter densities. Our results confirm that LSPA is superior to DWPDA in terms of tracking accuracy and dominates JPDA in terms of computation time. However, contrary to expectations, we found that the distance-weighting approach, when integrated with LSPA, does not enhance the performance of LSPA in terms of either tracking accuracy or computation time. The simulation scenarios in the experiment could be made more realistic with the addition of appearing and disappearing targets and time-varying target velocities. These scenarios add extra layers of complexity to the DA without affecting the conclusions we draw in this paper. Regardless, we demonstrated the validity of LSPA having computational requirements suitable for real-time processing and accuracy of tracking multiple targets in cluttered environments.

## Figures and Tables

**Table 1:** Average generalized optimal sub-pattern assignment (GOSPA) errors for tracking multiple crossing targets with clutter density $\lambda =3\times 10^{-4}/$m${}^{2}$.

	Number of Targets					
	1	2	3	4	5	6
LSPA	0.4571	0.9103	1.5033	1.9659	2.3441	2.9000
DWLSPA	0.4435	0.8787	1.5243	1.8601	2.2664	2.8253
JPDA	0.6751	2.5626	4.6425	6.1505	9.3851	11.5240
DWPDA	4.7454	9.1838	14.2879	18.5087	23.3401	27.4684

**Table 2:** Average GOSPA errors for tracking three targets with different clutter densities.

	λ				
	$1\times 10^{-4}$	$2\times 10^{-4}$	$3\times 10^{-4}$	$4\times 10^{-4}$	$5\times 10^{-4}$
LSPA	1.4280	1.6058	1.4056	1.6628	1.7690
DWLSPA	1.3718	1.5652	1.3388	1.5289	1.6257
JPDA	3.5367	4.2030	4.3475	4.4828	5.2677
DWPDA	7.4128	11.8464	14.4591	15.0590	15.8318

## Data Availability

No new data were created or analyzed in this study. Data sharing is not applicable to this article.

## Abbreviations

The following abbreviations are used in this manuscript:

DA	Data Association
MTT	Multiple Target Tracking
KF	Linear Kalman Filter
NN	Nearest Neighbor
PDA	Probabilistic Data Association
DWPDA	Distance-Weighting Probabilistic Data Association
PDAF	Probabilistic Data Association Filter
JPDA	Joint Probabilistic Data Association
JPDAF	Joint Probabilistic Data Association Filter
MHT	Multiple Hypothesis Tracking
SPA	Sum-Product Algorithm
LSPA	Loopy Sum-Product Algorithm
DWLSPA	Distance-Weighting Loopy Sum-Product Algorithm
LR	Likelihood Ratio
PDF	Probability Distribution Function
PMF	Probability Mass Function
NCV	Nearly Constant Velocity
RMS	Root Mean Square
GOSPA	Generalized Optimal Sub-Pattern Assignment
